# PON1 and Mediterranean Diet

**DOI:** 10.3390/nu7064068

**Published:** 2015-05-27

**Authors:** José M. Lou-Bonafonte, Clara Gabás-Rivera, María A. Navarro, Jesús Osada

**Affiliations:** 1 Departamento de Farmacología y Fisiología, Facultad de Ciencias de la Salud y del Deporte, Instituto de Investigación Sanitaria de Aragón-Universidad de Zaragoza, Huesca, E-22002, Spain; E-Mail: mlou@unizar.es; 2 CIBER de Fisiopatología de la Obesidad y Nutrición, Instituto de Salud Carlos III, Madrid, E-28029, Spain; E-Mails: clarisgr_@hotmail.com (C.G.R); angelesn@unizar.es (M.A.N.); 3 Departamento de Bioquímica y Biología Molecular y Celular, Facultad de Veterinaria, Instituto de Investigación Sanitaria de Aragón-Universidad de Zaragoza, Zaragoza, E-50013, Spain

**Keywords:** paraoxonase 1, PON1, Mediterranean diet, olive oil, nuts, fruits, nutraceuticals

## Abstract

The Mediterranean diet has been proven to be highly effective in the prevention of cardiovascular diseases. Paraoxonase 1 (PON1) has been implicated in the development of those conditions, especially atherosclerosis. The present work describes a systematic review of current evidence supporting the influence of Mediterranean diet and its constituents on this enzyme. Despite the differential response of some genetic polymorphisms, the Mediterranean diet has been shown to exert a protective action on this enzyme. Extra virgin olive oil, the main source of fat, has been particularly effective in increasing PON1 activity, an action that could be due to low saturated fatty acid intake, oleic acid enrichment of phospholipids present in high-density lipoproteins that favor the activity, and increasing hepatic PON1 mRNA and protein expressions induced by minor components present in this oil. Other Mediterranean diet constituents, such as nuts, fruits and vegetables, have been effective in modulating the activity of the enzyme, pomegranate and its compounds being the best characterized items. Ongoing research on compounds isolated from all these natural products, mainly phenolic compounds and carotenoids, indicates that some of them are particularly effective, and this may enhance the use of nutraceuticals and functional foods capable of potentiating PON1 activity.

## 1. Introduction

**Figure 1:**
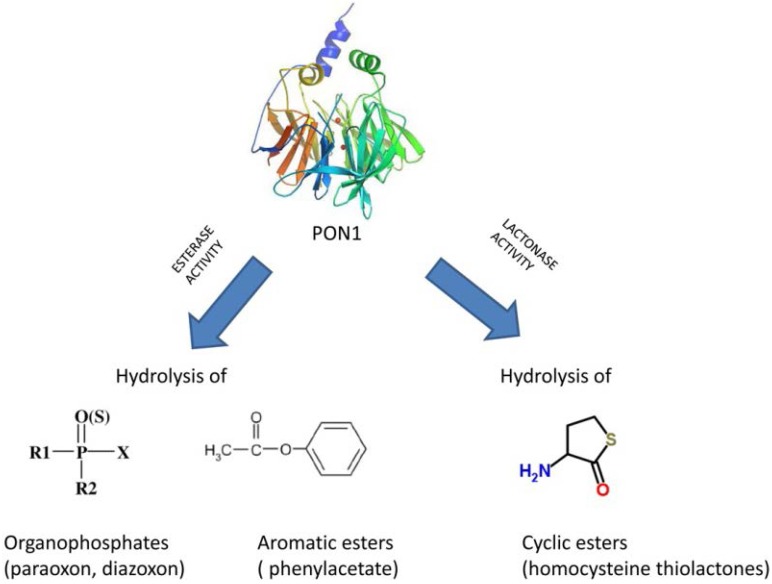
Paraoxonase 1 activities *in vitro*.

Human serum paraoxonase 1 (PON1), primarily synthesized by the liver, is mainly associated with serum high-density lipoproteins (HDL) [1] and, *in vitro*, displays a wide range of esterase activities (Figure 1). It has been reported to play a crucial role in the antioxidant activity of HDL by protecting low-density lipoproteins (LDL) against lipid peroxidation and, thus, attenuates the development of atherosclerosis [2]. In fact, PON1-deficiency resulted in increased oxidative stress not only in serum, but also in macrophages, a phenomenon that can contribute to the accelerated atherosclerosis shown in *Pon1*-deficient mice [3]. PON1 also exerts a protective effect against oxidative damage of cells and modulates the susceptibility of HDL and LDL to atherogenic changes such as homocysteinylation [4]. In addition, it modulates the anti-inflammatory role of HDL [5]. Moreover, low PON1 activity has been found in numerous pathological conditions associated with atherosclerosis, including type 1 and 2 diabetes, hypercholesterolemia and metabolic syndrome, as well as in elderly populations [6,7,8]. All these conditions display a pro-inflammatory baseline state that could be due to low PON1, as its activity is inversely related to cardiovascular risk [2,9].

**Figure 2:**
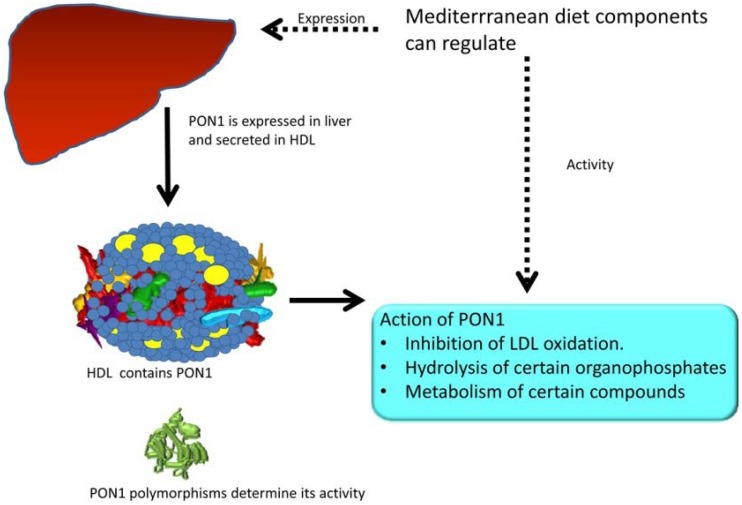
Effect of dietary components on regulation of paraoxonase 1 activity and its interaction with genetic factors. Adapted from [12] and [13], reproduced with permission from Elsevier.

The interindividual variation in PON1 activity is partly determined by the existence of genetic polymorphisms since human PON1 shows two major polymorphic isoforms, PON1-Q192R and PON1-L55M. The PON1-192 polymorphism is associated with diminished PON1 concentrations and an increased risk for coronary heart disease in RR-allele subjects [10]. PON1 L55M genotype (MM) frequency was significantly higher in those patients than in controls [11]. PON1 activity can be modulated by environmental conditions as well (Figure 2).

**Figure 3:**
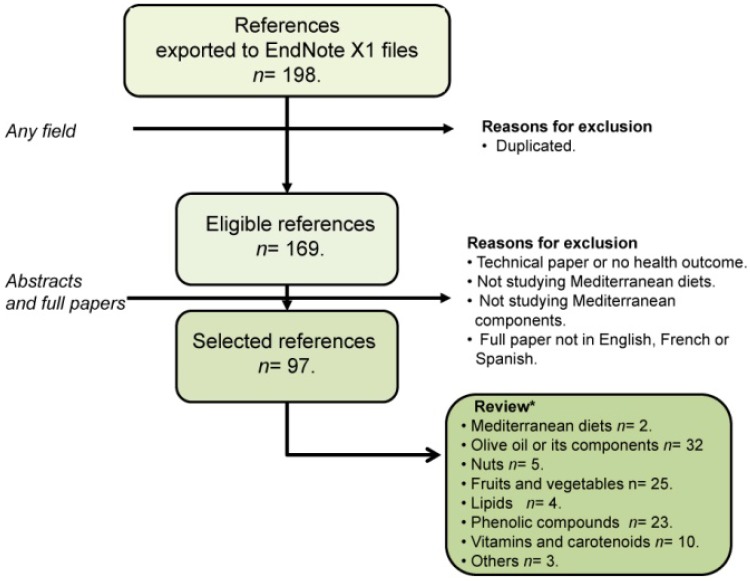
Flow chart displaying the stages used to select the references considered. EndNote X1 (Bld 2566, Thomson Reuters: New York, NY, USA, 2007). * Some references may appear in more than one section of the review.

A 2012 review [14] highlighted some of the more recent studies in the field of nutrigenetics at that time, which explored interactions among diet, genetic variation in antioxidant enzymes (including PON) and oxidative stress. It is, therefore, of interest to identify modulators of PON1 activity and concentration, and diet may be one of these factors [15]. The traditional Mediterranean diet used to be the food consumption pattern in countries like Spain, Italy, Greece and southern France during the decade of the sixties, and it has been associated with a lower incidence of coronary heart disease [16,17]. The Mediterranean diet is largely vegetarian in nature and includes the consumption of large quantities of olive oil as the main source of calories [18]. In the present paper, we will focus on the effect of the Mediterranean diet, its typical foods or chemical components and the effect of their intake on PON1 activity. To compile advances in the field, the present report has adhered to systematic review guidelines [19]. As displayed in Figure 3, a search in PubMed [20] using the keywords (PON1 and Mediterranean diet, nut and paraoxonase, olive oil and paraoxonase, food and paraoxonase as Mesh, resveratrol and paraoxonase, rutin and paraoxonase, carotenoids and paraoxonase, catechins and paraoxonase) identified 198 hits from November 1945 to March 11th, 2015. The search was refined by eliminating duplicate documents. The 169 papers obtained were critically reviewed to verify whether they analyzed PON1 and Mediterranean diet or its components. Documents that failed to meet this criterion were excluded. Thus, this review covers the works related to the effects of Mediterranean diet and PON1 in 97 papers.

## 2. PON1 and Mediterranean Diet

Two studies analyzed the effect of the Mediterranean diet as a whole on PON1 activity. In the first, paraoxonase activity was assayed in Greek migrants, who maintain a traditional Mediterranean diet, and compared with that of Anglo-Celtic individuals in Australia. Interestingly, paraoxonase activity correlated directly with circulating carotenoid concentrations in Greeks, and inversely with homocysteine and C-reactive protein in Anglo-Celtics. Such associations were seen only among subjects with the high activity phenotype (defined by the ratio of paraoxonase:arylesterase activity). The data suggest that dietary modulation of atherosclerotic risk may vary according to PON1 phenotype [21]. In the second study, the effect of two different fatty meals was addressed. A Mediterranean-like meal (61% monounsaturated fat) was compared to a Western-like meal (57% saturated fat), and only the former resulted in a significant increase in both PON1 activity and carotenoid concentrations [22]. These studies provide some evidence that the Mediterranean diet can alter PON activity.

## 3. PON1 and Olive Oil or Its Components

The importance of dietary lipids in paraoxonase activity was recently reviewed by Ferretti and Bacchetti [4] who, in line with other authors [23,24,25], propose that the amount and the composition of dietary lipids are key factors in the modulation of PON1. The effect of dietary lipids is also modulated by PON1 polymorphisms [26] and the molecular mechanisms involved include regulation of hepatic PON1 synthesis or secretion and/or modification of PON1 interactions with HDL [24]. Furthermore, changes in PON1 activity could also be related to dietary intake of oxidized lipids that behave as PON1 inhibitors [27].

From this perspective, there is a renewed interest in virgin olive oil as the main source of fat in the Mediterranean diet, especially first-press extra-virgin olive oil (EVOO), which retains minor compounds that may have antiatherosclerotic properties [28]. Human consumption of virgin olive oil lowers major atherosclerotic risk factors [29]. One proposed mechanism of action has been the increase in HDL levels [30]. However, the functionality of HDL may be as relevant to cardiovascular risk assessment as plasma HDL concentrations [31,32]. The anti-inflammatory activity of HDL has been attributed to apolipoprotein A1 (APOA1) and lipids, including sphingosine-1-phosphate and sphingosylphosphorylcholine [33,34]. However, there is strong evidence that PON1 also participates in this activity, acting alone or in combination with other HDL-associated enzymes to modulate the antioxidant and anti-inflammatory role of HDL [5,35]. Several studies have investigated whether EVOO consumption could improve the atheroprotective activity of HDL. In subjects consuming this oil for 12 weeks, the anti-inflammatory activity of both isolated HDL and PON1 was increased, the anti-inflammatory activity of HDL being modulated by PON1 [9]. Cherki *et al.*, also observed an increase in paraoxonase activity after the consumption of 25 mL/day of either argan oil or EVOO for two weeks, associated with an improvement in antioxidant status [36]. In a postprandial study, it was found that a single meal rich in olive oil raised PON1 arylesterase activity in the serum chylomicron fraction by 25% compared with a meal rich in cream [37], possibly due to the formation of a greater number of chylomicrons and delayed clearance of chylomicron remnants after ingestion of olive oil. A similar finding was observed when thermally stressed olive oil was given to middle-aged and older diabetic women [38]. These results point to a relationship between virgin olive oil consumption and PON1 levels in humans irrespective of experimental design.

The effect of olive oil has been studied in animal models as well. In this regard, in mice consuming fish, soy or olive oils, a significant decrease in postprandial serum PON1 activity was found only after fish oil supplementation, in accordance with the increase in serum oxidizability. PON1, in turn, attenuated postprandial oxidative stress after consumption of soy or olive oil, an action that was probably related to the effect of PON1 lipase-like activity on chylomicron triacylglycerols [39]. Also in animal models, the addition of cholesterol to chow or virgin olive oil diets significantly decreased APOA1 in *Apoe*-deficient females and serum paraoxonase activities in males. The latter activity was higher in females than in males. Size of aortic lesions was inversely correlated with circulating paraoxonase activity, particularly in males, and the relationship remained after adjusting for APOA1 and HDL cholesterol levels [40]. These results demonstrate that, together with sex, the nutritional regulation of paraoxonase is an important determinant of atherosclerotic lesions. When the comparison was established using different types of Western diets, no differences in plasma PON1 arylesterase activity or APOA1 concentrations were found in *Apoe*-deficient mice consuming EVOO from different cultivars (Arbequina, Picual, Cornicabra, or Empeltre) with respect to the diet containing palm oil. This fact suggests that the beneficial effects of EVOO could be due to the complex interactions of HDL particles. In fact, an elevated activity of PON1 was found in small, dense, protein-rich HDL. Despite the variation in arylesterase activity, no differences were found in APOA1, the classical PON regulator, a finding that rules out APOA1 as the inductor of this change in enzyme activity. This demonstrates that, even in a westernized diet scenario, cholesterol-poor, APOA4-enriched lipid particles generated by EVOO intake provide potent protection against oxidative stress, in part through increases in arylesterase activity [41]. The antioxidant activity of these small particles has been reported to be rather complex, with enzymatic or non-enzymatic involvement [42,43,44], or perhaps both. In BalbC mice consuming olive oil, there were increases in HDL phospholipids/protein, HDL-PON1 arylesterase activity and the PON1 contribution to HDL-mediated macrophage cholesterol efflux. Therefore, a phospholipid-stimulatory effect on PON1 was proposed. A mutant PON1 lacking the first 20 amino acids, generated by direct mutagenesis, was unable to display the phospholipid stimulatory effect, indicating that this protein domain is critical for the interaction of PON1 with phospholipids and that this interaction is crucial in the olive oil-induced effect [45].

Oleic acid (18:1) is the major fatty acid present in olive oil (Table 1). Due to this fact, it has been proposed that its intake could determine olive oil-induced changes in PON1 by replacing saturated fat and limiting the negative consequences of the latter on this enzyme [24,25]. In this respect, a population-based study showed that high oleic acid intake was associated with increased HDL cholesterol levels and PON1 activity only in subjects with the QR and the RR genotypes of the PON1-192 polymorphism, respectively [26]. Thus, the beneficial effect of increasing oleic acid intake on HDL and PON1 activity would be more pronounced in subjects carrying the R allele. Likewise, olive oil consumption by healthy subjects increased HDL-PC-18:1 levels and HDL-PON1 activities [45]. However, other minor components of olive oil (Table 1) have also been shown to promote changes in PON1 activity. In this respect, Hussain *et al.*, observed decreased hepatic paraoxonase activity in rats with experimental non-alcoholic fatty liver disease consuming unmodified olive oil [46]. However, in a study of senescence-accelerated mouse-prone 8 (SAMP8) mice fed a diet enriched in 10% olive oil containing either high or low amounts of olive oil phenolics for 4.5 months, the results were higher serum PON1 activity and *Pon2* mRNA levels in heart tissue of animals consuming the high-phenolic diet. This effect could be attributable to the olive oil phenolic compound, hydroxytyrosol, which may be responsible for the induction of *Nrf2*-dependent gene expression and the increase in PON activity [47]. Supplements of EVOO with green tea polyphenols given to *Apoe*-deficient mice also resulted in an increase in serum paraoxonase 1 [48]. Nonetheless, when male *Apoe*-deficient mice were fed pure hydroxytyrosol at a dose of 10 mg/kg/day for 10 weeks, changes in serum paraoxonase were not observed. These data support the concept that phenolic-enriched products taken out of the original matrix may be not useful [49]. Something similar might occur with squalene (the main hydrocarbon in the unsaponifiable fraction of olive oil). When administered in a glycerol solution, it did not elicit any change in paraoxonase activity in *Apoe*-deficient mice of either sex [50]. However, the same dose of squalene, but in a different administration regimen (dissolved in the oil fraction of chow diet), promoted changes in HDL-cholesterol and PON1 and decreased reactive oxygen species in lipoproteins and plasma malondialdehyde levels, not only in *Apoe*-deficient mice, but also in wild-type and *Apoa1*-deficient mice [51]. The importance of the lipid matrix has also been observed when studying PON1 using high-phenolic preparations of coconut oil [52]. These results indicate that minor components present in EVOO may participate in the increase in PON1 activity, but show a special dependence on the triglyceride matrix present in olive oil. Overall, these findings support an important role for olive oil in PON1 activity, a circumstance that could partly explain the beneficial effect of the Mediterranean diet on health status.

**Table 1:** Composition of virgin olive oils.

Component	Content (g%)
**Fatty Acids of Triglycerides**	
Myristic (14:0)	0.0–0.05
Palmitic (16:0)	7.5–20
Palmitoleic (16:1n7)	0.3–3.5
Margaric (17:0)	0–0.3
Heptadecenoic (17:1)	0.0–0.3
Stearic (18:0)	0.5–5.0
Oleic (18:1n9)	55–83
Linoleic (18:2n6)	3.5–21
α-linolenic (18:3n3)	0.0–0.9
Arachidic (20:0)	0.0–0.6
Eicosenoic (20:1n9)	0.0–0.4
Behenic (22:0)	0.0–0.2
Lignoceric (24:0)	0.0–0.2
**Minor Components**	
Terpene compounds	0.1–0.3
Phytosterols	0.1–0.2
Hydrocarbons	
Squalene	0.1–0.8
Carotenes	0.05–0.1
Phenolic compounds	0.05–0.1

Adapted from [53,54,55,56,57]. Reproduced with permission from Wiley and Sons

## 4. PON1 and Nuts

Several epidemiological studies suggest that consumption of nuts promotes a healthy lipid profile associated with a lower risk of cardiovascular disease. These studies have attributed this quality to the nutrients they contain, mainly monounsaturated and polyunsaturated fatty acids and many other bioactive constituents, such as antioxidants, phytosterols and other phytochemicals [58,59]. Dietary interventions aimed to prove the effects of nuts on PON1 have been carried out in humans and in animal models. The former approach involved subjects at increased cardiovascular risk in whom a walnut paste-enriched meat improved the antioxidant status [60,61] and significantly lowered plasma levels of cell adhesion molecules (sVCAm-1 and sICAM-1) and leukotriene B4, compared to low-fat meat. These changes seemed to be related to PON1 activity, and most of the variability could be explained by PON1 and APOA4 polymorphisms [62,63]. However, providing Brazil nuts to normolipidemic individuals at a dose of 45 g per day for 15 days did not modify PON1 activity [64]. In view of these discrepancies, dose may be an important issue. In this regard, while a 2.5 g/day pistachio intake increased serum paraoxonase activity in rats compared with a standard diet, a larger amount of 5 g/day was ineffective [65]. The influence of sex cannot be disregarded and, in this respect, in *Apoe*-deficient mice, supplementation (30 g/day) with a mixture of three nuts (50% walnuts, 25% almonds and 25% hazelnuts) for 12 weeks raised PON1 activity and increased hepatic *Pon*2 expression only in females, when compared to those receiving an isoenergetic diet containing palm oil [66]. Overall, these results suggest that the effect of nuts on PON1 may vary according to the varieties consumed, a circumstance that might be influenced by the balance of saturated/unsaturated fatty acids and other accompanying constituents. More studies are required to establish the dose and the role of sex in the response observed.

## 5. PON1 and Other Constituents of Mediterranean Diet

### Fruits and Vegetables

An important feature of the Mediterranean diet is the high intake of fruits and vegetables. Epidemiological studies have shown a relationship between a reduced risk of cardiovascular disease and fruit consumption [67,68]. Large prospective cohort studies have revealed that a high dietary intake of fruits and vegetables reduces cardiovascular disease risk [67,69]. In a clinical trial in subjects with type 2 diabetes, increased fruit and vegetable consumption augmented the carotenoid concentrations and PON1 associated with the antioxidant properties of HDL [70]. Much of this protective effect has been attributed to the biological activity of the non-nutrient secondary plant metabolites such as flavonoids and diverse phenolic compounds, which, in general, are powerful antioxidants [71]. Intake of antioxidant-rich foods, like fruit and vegetable juices, affect the activity and/or concentration of PON1 [15]. In diabetic patients from two European countries, a flavonoid-rich diet was positively associated with PON1 arylesterase activity [72]. However, in healthy volunteers with adequate vitamin intake, six week diets differing markedly in the amounts of linoleic and oleic acid and vegetables, berries and apples did not differ in their effects on lipid peroxidation or lipoprotein metabolism, and all these diets decreased PON1 activity [73]. Similarly, in nonsmoking women, two diets, one low and the other high in vegetables and having some differences in fatty acid contents, showed that the serum PON1 activity was lower after the high vegetable compared with the low vegetable diet. The reduction of PON1 activity correlated with the reduction in HDL cholesterol. High baseline PON1 activity was related to the presence of the R- and L-alleles of the PON1-192 and PON1-55 genotypes, respectively [74]. These discrepant results evidence a complex picture in which source of fruits and their composition, dietary fat content, special population needs and genetic PON1 isoforms are interacting and, therefore, different outcomes are possible.

Pomegranate has attracted much research interest as a source of some potent phenolic antioxidants (tannins, anthocyanins) whose role in protecting LDL and HDL from lipid oxidation has been clearly demonstrated [75]. This role involves two mechanisms: a direct interaction of pomegranate phenolics with LDL and an indirect effect by which they increase HDL-associated PON1 [76] and augment PON1 synthesis in the liver [77]. The enhancement of PON1 activity by pomegranate was clearly demonstrated *in vivo* in animal models and in humans, either healthy subjects or patients with diabetes or carotid artery stenosis. First, apolipoprotein E-deficient mice with advanced atherosclerosis that received pomegranate supplementation for two months showed a significant reduction of atherosclerotic lesions. This result may be attributed to the protection of LDL against oxidation and the significant increase in serum paraoxonase activity [78,79]. Using C57BL/6 control mice, *Pon1*- or *Pon2*-deficient mice, Rosenblat *et* *al.*, showed that pomegranate juice consumption also had an antioxidant effect on mouse macrophages that was mediated via stimulation of macrophage *Pon2* expression and was independent of PON1 [75]. Secondly, in healthy subjects, serum PON1 arylesterase activity was significantly increased after pomegranate juice consumption for a period of two weeks [80]. Even one week of pomegranate consumption was sufficient to induce serum PON1 activity. This effect was comparable to administration of black currant juice [81]. Thirdly, in diabetic patients, PON1 activity increased significantly by 12% following consumption of pomegranate juice for three months [82]. In another group of patients with type 2 diabetes, administration of 200 mL of pomegranate juice for a shorter period (six weeks) induced similar effects on PON1 activity [83]. In addition, four weeks of Wonderful variety pomegranate juice consumption significantly increased HDL-associated PON1 activity, reversing the dissociation between HDL and PON1 observed in these patients. Association of PON1 with HDL stabilizes the enzyme and stimulates its catalytic activity [84]. Similar results were observed, although to a lesser extent, with administration of an extract of pomegranate peel. Furthermore, *in vitro* incubation of serum from diabetic patients with pomegranate juice or its purified major phenolic compounds, such as punicalagin, gallic acid and ellagic acid, showed that all of them increased PON1 binding to HDL [85]. Finally, a time-dependent effect of pomegranate consumption on PON1 was observed in patients with carotid artery stenosis. By 12 months, PON1 activity had increased by 83% over the initial level [86].

Much less attention has been devoted to other fruits, whether in preclinical or clinical settings. In an animal model involving Syrian hamsters, consumption of raspberry juice for 12 weeks enhanced PON1 activity if expressed as a ratio to HDL in [87]. Using a different approach, administration of apple juice to a group of male Wistar rats for 28 days protected against the decrease in PON1 activity induced in another group of male Wistar rats that had received no juice when *N*-nitrosodiethylamine or carbon tetrachloride was given to both groups of animals 24 h before samples were taken. In fact, in the juice-pretreated rats, PON1 activity was actually enhanced despite the administration of the toxicants [88]. In human studies, different groups of patients have been recruited. When the effect of 28-day intake of antioxidant-rich orange and blackcurrant juices (with or without vitamin E supplement) on PON1 activity was explored in patients with peripheral arterial diseases, no significant changes were observed. Despite these results, there was a potential gene-diet interaction since subjects carrying the PON1 L55-allele showed increased PON1 activity following juice consumption without vitamin E [89]. Likewise, although a short intervention period (two weeks) of a low carotenoid diet with tomato or carrot juice failed to affect PON1 activity in healthy volunteers, tomato juice intake reduced lipid peroxidation in subjects carrying the R-allele of the PON1-192 genotype [90]. However, PON1 activity increased in elderly subjects consuming tomato juice, and those who were R-allele carriers were high responders [10]. Administration of lycopene, the carotenoid present in tomato juice, at 224–350 mg/week increased PON1 activity in serum and HDL in moderately overweight middle-aged subjects [91]. In healthy subjects, date consumption decreased basal serum oxidative status and lipid peroxidation, and increased paraoxonase1 activity. Dates are fruits typical of some countries of the Mediterranean basin that possess different concentrations of phenolic compounds (phenolic acids, mainly ferulic acid and coumaric acid derivatives, as well as chlorogenic and caffeic acid derivatives, catechins and a quercetin derivative as well) [92]. These findings suggest differential responses among genotypes and the need to improve identification of chemical compounds provided by the different fruits.

## 6. PON1 and Chemical Compounds Present in Mediterranean Diet and Their Potential as Nutraceuticals

As pointed out above, often controversial findings regarding the impact of Mediterranean diet constituents on PON1 have raised concern as to the extent of the influence of the complex mixture of chemical compounds on efforts to delineate the most relevant ones and to use them as potential nutraceuticals able to enhance PON1 activity. In this section, the works studying the effect of various compounds present in the Mediterranean diet on PON1 expression/activity are categorized and analyzed.

### 6.1. Lipids

Several approaches have been used to test the effect of a number of fatty acids on PON1. In this way, to explore the effect of oleic acid, *in vitro* incubation of serum with di-oleoyl-phosphatidylcholine was carried out, resulting in its incorporation into HDL with a consequent increase in PON1 activity. Furthermore, the contribution of PON1 to HDL-mediated cholesterol efflux from macrophages was greater in di-oleoyl-phosphatidylcholine-enriched HDL in comparison to control HDL [45]. However, administration of a conjugated linoleic acid isomeric mixture (4.5 g/day) for four weeks did not change paraoxonase as compared with safflower oil, but significantly reduced its arylesterase activity in overweight men [93]. Fish oil supplements tested in female patients with rheumatoid arthritis increased serum HDL-C, PON1 levels and arylesterase activity [94]. However, when 2 g/day of purified eicosapentaenoic/docosahexaenoic acids were given to individuals with type 2 diabetes mellitus for six weeks, no significant differences in fasting and postprandial circulating paraoxonase-1 activity were observed [95]. These results seem to indicate that not all fatty acids behave in similar ways regarding PON1, and that oleic acid displays a positive action.

### 6.2. Phenolic Compounds

Quercetin is a flavonol found in many fruits, vegetables, leaves and grains present in the Mediterranean diet. In this regard, dietary quercetin supplementation (2 mg/g diet) for six weeks induced hepatic *Pon1* gene expression with a tendency for greater induction in APOE3 as compared to APOE4 mice, suggesting that PON1 is differentially regulated in response to APOE genotype [96]. However, most dietary quercetin comes from rutin (quercetin-3-*O*-rutinoside). Rutin is a citrus flavonoid glycoside found in many constituents of the Mediterranean diet (especially the citrus fruits, peaches and apples). Rutin is metabolized by intestinal bacteria [97], releasing quercetin. Al-Rejaie *et al.*, reported that dietary rutin increased the expression of the hepatic *Pon1* gene, among other antioxidant genes in hypercholesterolemic male Wistar rats [98].

Anthocyanin is also a flavonoid present in many Mediterranean diet foods (*i.e.*, eggplant, blackberries, plums, grapes, red wine, pomegranates and cherries) [99]. Anthocyanin supplementation (160 mg twice daily for 24 weeks) increased HDL cholesterol, the activity of HDL-PON1 and cholesterol efflux capacity compared with placebo in hypercholesterolemic subjects. Furthermore, HDL-PON1 activity was negatively correlated with HDL lipid hydroperoxides and positively with cholesterol efflux. Based on these findings, anthocyanin may prove to be a cardioprotective nutraceutical [100]. Isoflavones, genistein and daidzein, are also present in some foods of the Mediterranean diet, *i.e.*, fava bean (Vicia faba) and lupine (*Lupinus* spp.) [101]. Three studies have addressed the influence of dietary isoflavonoids on PON1 activity. In the first, Ustundag *et al.*, studied the protective effect of soy isoflavones (100 mg/kg in diet for eight weeks) on experimental non-alcoholic steatohepatitis and their effects on plasma paraoxonase and arylesterase levels in rats, concluding that soy isoflavones seemed to be effective in preventing liver damage by increasing paraoxonase activity and decreasing lipid peroxidation [102]. In the second study, Mohammadshahi *et al.* observed that genistein or daidzein reversed the arthritis-induced decreases in paraoxonase and arylesterase activity in rats [103]. In the third study, Schrader *et al.* screened different flavonoids for their ability to induce PON1 in Huh7 cultured hepatocytes. Genistein was the most potent flavonoid with regard to its PON1-inducing activity, followed by daidzein, luteolin, isorhamnetin and quercetin. Other flavonoids such as naringenin, cyanidin, malvidin and catechin showed little or no PON1-inducing activity. In contrast, when these authors tried to reproduce the genistein effect *in vivo* by administering this flavonoid to growing rats at a dose of 2 g/kg diet over three weeks, the increases in hepatic *Pon1* mRNA and protein levels and in plasma PON1 activity were not reproduced [104]. Catechins deserve special attention since they are present in Mediterranean foods (*i.e.*, peaches, barley grain, wine and vinegar) [105,106,107]. Different experimental models involving toxins or metabolic diseases (*i.e.*, HgCl_2_ toxicity [108], ethylene glycol-induced renal failure [109], streptozotocin-induced diabetic rats [110], *Apoe*-deficient mice [111,112] and cystathionine beta synthase-deficient mice [113]); catechins, alone or in combination with other polyphenols (quercetin) or foods (wine); or procyanidins (a group of flavonoids that are oligomeric forms of catechins present in red wine, grapes and apples) [110] have demonstrated up-regulation of PON1 levels or protective activity against LDL oxidation and lipid peroxidation. These studies raised important caveats such as the influence of the chemical nature of flavonoids or isoflavonoids, the experimental approach and selection of the animal model.

Curcumin is a phenolic compound obtained from the root of *Curcuma longa* and a common spice in the Mediterranean diet. Curcumin supplementation (0.05% in diet) of a high-fat diet significantly elevated plasma HDL cholesterol, APOA1 and paraoxonase activity compared with the control group in hamsters [114]. Moreover, curcumin dose-dependently induced PON1 transactivation in Huh7 cells. However, dietary supplementation with curcumin (500 mg/kg diet) for two weeks in female B6C3F1 mice did not modify the hepatic PON1 mRNA or protein level [115]. In conclusion, curcumin is a potent PON1 inducer in cultured cells *in vitro*, but its effect *in vivo* may be influenced by the animal model or dietary fat content.

Resveratrol is a naturally occurring phenolic compound that is present at high levels in wine. *In vitro*, resveratrol has been shown to enhance PON1 enzyme activity and gene expression in hepatocytes, HepG2, HC04 and A549 cells [116,117,118]. This effect could be mediated by the resveratrol activation of the aryl hydrocarbon receptor as a putative inducer of PON1 [119,120]. More controversial have been the outcomes of resveratrol administration *in vivo*. Whereas chronic administration of resveratrol significantly decreased serum paraoxonase-1 activity in cystathionine beta synthase-deficient mice [121], in *Apoe*-deficient mice, serum paraoxonase activity was significantly higher in the resveratrol group [122]. Resveratrol, as other phenolic compounds, is a potent PON1 inducer in cultured cells, but its effect *in vivo* may be influenced by the animal model or dietary regimen.

### 6.3. Vitamins and Carotenoids

As was pointed out previously, the Mediterranean dietary pattern is characterized by abundant consumption of fruits and vegetables, which are a good source of all types of vitamins and carotenes. For instance, vitamin E is present in many constituents of this diet (e.g., olive oil, nuts and seeds) and vitamin C in citrus fruits and vegetables. Vitamin E supplementation reversed the decreased serum paraoxonase and arylesterase activities in hypothyroidism induced by propylthiouracil administration to rats [123]. Similarly, vitamin E supplementation for 10 weeks had a protective effect against the decrease in PON1 activity observed in exercise-induced oxidative damage in sedentary dogs [124]. Moreover, the decreased post-exercise PON1 activity was normalized by alpha-tocopherol supplementation in basketball players, [125]. Vitamin C administration restored the decreased serum PON1 activity of hemodialysis patients [126]. The incorporation of vitamin A into the diet of vitamin-A-deficient rats reverted the decreased serum paraoxonase1 induced by vitamin A deficiency [127]. These reports point to a role of vitamins A, E and C in restoring the decreased paraoxonase activity following different oxidative stress-causing conditions.

As was mentioned above, carotenoids are highly present in the Mediterranean diet and may play a role in PON1 activity. Indeed, incubation with β-carotene counteracted the IL-1β-induced decrease in PON1 expression in human endothelial cells. The effect of β-carotene was reversed by a calmodulin-dependent protein kinase kinase inhibitor, a finding that indicates a role of this pathway in PON1 expression [128]. Astaxanthin is a β-carotene-like carotenoid pigment present in crustaceans and salmon, but it is also a metabolite of zeaxanthin that is found in paprika, peppers and leaves of green vegetables, many of them constituents of the Mediterranean diet. Astaxanthin supplementation for 90 days increased PON1 activity in soccer players, an effect that could be due to the protection of free thiol groups against oxidative modification observed during the treatment [129]. Lycopene, a carotenoid present in tomatoes, also displayed a restorative effect upon the decreased PON1 activity observed in rats subjected to experimental diabetes [130]. In moderately overweight middle-aged individuals, McEneny *et al.* found that lycopene supplementation increased PON1 activity in serum and HDL [91]. However, it has been reported that PON1 polymorphisms may modify the association between serum concentrations of lycopene and oxidative stress parameters [131]. According to these findings, carotenes exert an important effect on PON1, while other potential mechanisms are likewise emerging.

### 6.4. Coenzyme Q_10_

CoenzymeQ_10_ is synthesized in most human tissues, and the enzyme hydroxymethylglutaryl-CoA reductase plays a critical role in its regulation [132]. Rich sources of dietary coenzyme Q_10_ include mainly meat, poultry, fish and nuts. Fruits, vegetables, eggs, and dairy products are moderate sources of this coenzyme [133,134,135]. Coenzyme Q_10_ exerts a protective effect on plasma lipoprotein oxidation. Administration of extra virgin olive oil (20 mL) enriched with either 20 or 40 mg of the coenzyme for two weeks induced a dose-dependent increase in paraoxonase-1 activity in healthy subjects [136]. These findings indicate that there is still a potential to improve the quality of the influence of extra virgin olive oil on PON1.

### 6.5. Taurine

Good sources of dietary taurine are seafood and meat [137]. Administration of taurine to rats with hypothyroidism resulted in a dose-dependent increase in serum paraoxonase and arylesterase activities [138]. This observation prompts the notion that certain amino acids may be particularly active in regulating PON1 expression.

### 6.6. Trace Elements

Selenium is present in components of the Mediterranean diet such as seafood (mussels), fish (tuna), whole-wheat bread, and seeds (sunflower). Selenium supplementation in male Sprague Dawley rats improved the high fat diet-mediated reduction of serum PON1 enzyme activity by 34% and PON1 protein levels by 21%, with no changes in its hepatic mRNA expression [139]. Thus, this supplementation could be a valuable approach to limiting the adverse effects of high fat diet-induced hypercholesterolemia and may warrant further investigation.

## 7. Conclusions

One of the key actions of the Mediterranean diet is the prevention of cardiovascular diseases, which can be promoted in part by increased PON1 activity. This hypothesis is supported by the increase in this enzyme when Mediterranean diets have been administered. A dissection of the components of these diets points to EVOO intake as an important factor of PON1 levels. It is noteworthy that some PON1 polymorphisms are crucial determinants of EVOO response. Not only is EVOO consumption replacing intake of noxious saturated fatty acids, but it also contributes to establishing the phospholipid environment of HDL that enhances PON1 activity. In addition, some minor components of EVOO increase hepatic PON1 protein and mRNA expressions. An emerging role of nuts and their components in the activity of this enzyme has also been observed. Likewise, fruits and vegetables are able to modify this activity, the pomegranate and its compounds being well characterized examples. The characterization of isolated compounds from all these and other natural sources, such as phenolic compounds, carotenoids, *etc.*, will lead the way to interesting nutraceuticals and the development of functional foods to enhance the protective role of PON1 (Table 2).

**Table 2:** Overview of the studies reviewed.

	Characteristics of Studies	Findings	References
Mediterranean diets	Greek tradition *vs.* Anglo-Celtics	Paraoxonase activity correlated with carotenoid concentrations	[21]
	Mediterranean-like meal was compared to a Western-like meal	Increase in PON1 activity and carotenoid concentrations	[22]
Olive oil or its components	Virgin olive oil in humans	Increased PON1 levels	[9,36,37]
	Olive oil in animal models	Favors PON1	[39,40,45]
	Oleic acid intake in humans	The beneficial effect on PON1 activity was dependent on polymorphisms	[26]
	Olive oil and green tea phenolics in animals	Increased PON1 activity	[47,48]
	Squalene	Variable effects depending on matrix vehicle	[50,51]
Nuts	Human and animal studies	Effect on PON1 may vary according to different nuts and their constituents	[62,63,64,65,66]
Fruits and vegetables	Increased consumption in humans	Augmented PON1 with some fruits and phenotypes	[15,70,71,72,73,74,76,89,90,91,92]
Lipids	Human and animal studies	Differential effects depending on different fatty acids	[45,93,94,95]
Phenolic compounds	Quercetin in mice	PON1 differentially regulated depending on APOE genotype	[96]
	Anthocyanin in humans	Increased HDL-PON1	[100]
	Flavonoids and isoflavones	Discrepant results in function of experimental approach	[101,102,103,104,108,109,110,111,112,113]
	Curcumin	*In vivo* effect is influenced by the animal model and dietary fat content	[114,115]
	Resveratrol	Animal model and dietary regimen modify the outcome	[116,117,118,119,120,121,122]
Vitamins and carotenoids	Vitamin A, C and E supplementation	Positive action on PON1	[123,124,125,126,127]
	β-carotene, astaxanthin, lycopene	Increased PON1 activity	[91,128,129,130,131]
Coenzyme Q_10_	Humans consuming olive oil enriched with this compound	Increase in PON1 activity	[136]
Taurine	Rats with hypothyroidism	Increase in serum paraoxonase	[138]
Trace elements	Selenium supplementation to rats	Increase in serum paraoxonase	[139]
